# A composite scaffold of Wharton’s jelly and chondroitin sulphate loaded with human umbilical cord mesenchymal stem cells repairs articular cartilage defects in rat knee

**DOI:** 10.1007/s10856-021-06506-w

**Published:** 2021-03-29

**Authors:** Zhong Li, Yikang Bi, Qi Wu, Chao Chen, Lu Zhou, Jianhong Qi, Di Xie, Hongqiang Song, Yunning Han, Pengwei Qu, Kaihong Zhang, Yadi Wu, Qipu Yin

**Affiliations:** 1 Institute of Sports Medicine, Shandong First Medical University & Shandong Academy Medical Sciences, 619 Changcheng Road, Taian, 271016 Shandong PR China; 2 Clinical Center for Sports Medicine and Rehabilitation, the Affiliated Hospital of Shandong First Medical University, 706 Taishan Great Street, Taian, 271000 Shandong PR China

## Abstract

To evaluate the performance of a composite scaffold of Wharton’s jelly (WJ) and chondroitin sulfate (CS) and the effect of the composite scaffold loaded with human umbilical cord mesenchymal stem cells (hUCMSCs) in repairing articular cartilage defects, two experiments were carried out. The in vitro experiments involved identification of the hUCMSCs, construction of the biomimetic composite scaffolds by the physical and chemical crosslinking of WJ and CS, and testing of the biomechanical properties of both the composite scaffold and the WJ scaffold. In the in vivo experiments, composite scaffolds loaded with hUCMSCs and WJ scaffolds loaded with hUCMSCs were applied to repair articular cartilage defects in the rat knee. Moreover, their repair effects were evaluated by the unaided eye, histological observations, and the immunogenicity of scaffolds and hUCMSCs. We found that in vitro, the Young’s modulus of the composite scaffold (WJ-CS) was higher than that of the WJ scaffold. In vivo, the composite scaffold loaded with hUCMSCs repaired rat cartilage defects better than did the WJ scaffold loaded with hUCMSCs. Both the scaffold and hUCMSCs showed low immunogenicity. These results demonstrate that the in vitro construction of a human-derived WJ-CS composite scaffold enhances the biomechanical properties of WJ and that the repair of knee cartilage defects in rats is better with the composite scaffold than with the single WJ scaffold if the scaffold is loaded with hUCMSCs.

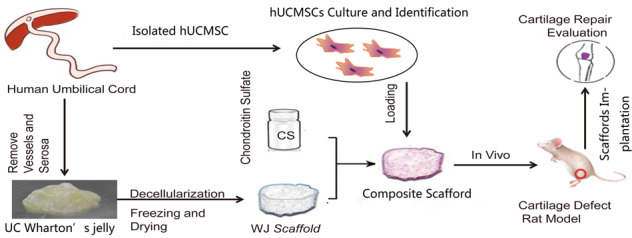

## Introduction

The repair and treatment of full-thickness articular cartilage injury remains a challenge in orthopedic surgery and sports medicine [1]. The rapid development of cartilage tissue engineering technology has provided new ideas for the repair of cartilage defects [2]. The strategies of tissue engineering commonly include the application of combinations of biomaterials, cells, and biologically active factors to form tissue replacements [3]. Many studies have shown that mesenchymal stem cells (MSCs) can be used as seed cells for repairing cartilage injuries in animals [4–6]. Bone marrow-derived MSCs have long been the gold standard for bone and cartilage tissue engineering [7]. However, bone marrow MSCs face problems such as the low yield of single-pumped stem cells, difficulty in culture expansion, donor infection, and differentiation potential inversely proportional to age [8] and limited use. In contrast, umbilical cord (UC)-derived MSCs have desirable characteristics. First, the UC, which is discarded at birth, provides an inexhaustible source of stem cells for therapy. Furthermore, human UC mesenchymal stem cells (hUCMSCs) have faster proliferation rates and greater expansion capability than adult MSCs and possess broad multipotency without inducing teratomas [9, 10]. The efficacy of hUCMSCs in cartilage repair has been demonstrated in animal studies [8, 11, 12], and hUCMSCs are thus prospective cell candidates for tissue-engineered cartilage.

The construction of engineered tissues is often based on the structures of scaffold biomaterials that support the function and growth of new tissue. Both the material and manufacturing process must be considered to ensure that the mechanical and physiological properties of the de novo tissue closely match those of the native tissue [13]. Increasing levels of complexity in repairing tissues or organs are generally consistent with a concomitant increase in the complexity of the associated tissue engineering approach [3]. In recent years, Wharton’s jelly (WJ) has been found to have components similar to those of articular cartilage and contain some chondrogenic growth factors, such as insulin-like growth factor I and transforming growth factor-*β*. Studies have shown that cartilage tissue engineering scaffolds prepared with WJ can effectively repair articular cartilage defects [14, 15, 17]. Thus, WJ has become a promising raw material for tissue-engineered cartilage [16–18]. However, its physical properties, such as modulus of elasticity, usually do not satisfy the requirements of cartilage repair. These properties may be modified by introducing intermolecular crosslinks [19]. Chondroitin sulfate (CS), with a relative molecular mass ranging from 10,000 Da to 50,000 Da, is an acidic polysaccharide composed of disaccharide units of glucuronic acid and aminohexose. CS distributed in various animal tissues has been demonstrated to possess various biological activities, such as adjusting immunity and fat metabolism, preventing infection, and reducing the risk of heart diseases [20]. CS has broad application prospects in the fields of medicine, food and cosmetics, and the use of CS in the preparation of bioengineered scaffolds can improve the physical characteristics of the scaffold material [21, 22].

According to the requirements of tissue-engineered scaffolds, does the scaffold composite prepared by crosslinking WJ with CS have greater biomechanical performance? Does the composite scaffold containing WJ and CS have a better repair effect in vivo than the WJ scaffold alone? No relevant reports that answer these questions have been published. In this study, a novel composite scaffold was prepared with a WJ scaffold, its performance was tested in vitro, and the cartilage defect of a rat knee was repaired in vivo with different scaffolds loaded with hUCMSCs. The repair effect was assessed by gross appearance and histological grading scores. This study provides an experimental basis for the clinical feasibility of tissue-engineered cartilage.

## Methods and materials

### Isolation, culture and identification of hUCMSCs in vitro

All experimental protocols were approved by the Medical Ethics Committee of Shandong First Medical University & Shandong Academy Medical Sciences (No. 201758). Human UCs were collected aseptically from normal full-term births from the Maternity Department at the Affiliated Hospital of Shandong First Medical University & Shandong Academy Medical Sciences after informed consent was obtained. The cords were rinsed twice with phosphate-buffered saline (PBS) (Solarbio, Beijing, China), and the cord blood was cleaned in penicillin and streptomycin. The washed cords were cut into 1.0–2.0 mm sized pieces and suspended in Dulbecco’s modified Eagle’s medium (Gibco, Life Technologies, USA) containing 10% fetal bovine serum (BI, Israel), 5% horse serum (Gibco, USA), penicillin and streptomycin. The cord pieces were then incubated at 37 °C in a humidified atmosphere with 5% CO_2_ under normoxic conditions. The nonadherent cells were then washed off. The medium was changed every 3 days. When well-developed colonies of fibroblast-like cells appeared after 10 days, the cultures were trypsinized (without dilution) and transferred into a new vessel for further expansion; the medium was replaced every 3 days. To detect the typical markers of different passages of hUCMSCs, third-generation hUCMSCs were selected.

Immunofluorescence double staining was used to identify the phenotype of the hUCMSCs. A suspension of third-generation hUCMSCs was prepared after digestion with a cell digestion solution in 0.25% trypsin (Beijing Solei Chemical Co., Beijing, China) and added dropwise into six-well plates. The cells were incubated at 37 °C in 5% CO_2_ for 6 h. The samples were fixed in paraformaldehyde for 10 min, permeabilized with Triton X-100 (Amresco, USA) for 5 min and then incubated with 1% bovine serum albumin (BI Company, Israel) for 1 h. Anti-CD44 at a 1:1000 dilution and anti-CD90 at a 1:200 dilution (Abcam, UK) were added, and the moisture *of the solutions was* preserved during the overnight incubation at 4 °C. The cells were incubated in PBS for 50 min before the addition of the secondary antibody (DAKO, Denmark). Then, after they were washed in PBS, the cells were stained with 4′,6-diamidino-2-phenylindole (DAPI) for 5 min and then observed under a fluorescence microscope (Nikon D90, Japan).

### Construction of WJ scaffolds in vitro

The UC tissues were cut open from the center under sterile conditions, and then the outer tissue and vascular tissue were peeled away. The remaining adhesive tissues (WJ) were rinsed with sterile distilled water for 5 min; this procedure was repeated 3 times. The tissues were subsequently sterilized in 3% H_2_O_2_ for 30 min and washed with sterile distilled water three times for 30 min each time. The jelly was placed in a grinder with three volumes of sterile distilled water and repeatedly crushed into a homogenate at −5 °C. Then, after adding five volumes of sterile distilled water, the homogenate was frozen at −20 °C and then thawed at room temperature. This freeze-thaw cycle was repeated four or five times. The homogenate was centrifuged using various centrifugation steps (Beckman Allegra X-22R, USA) for 20 min at 3000 rpm, after which the supernatant was removed from the homogenate. Then, the supernatant was centrifuged for 20 min at 6000 rpm and separated again by gradient centrifugation for 30 min at 7000 rpm. Afterward, it was separated again by gradient centrifugation for 30 min at 10,000 rpm. The final supernatant was discarded, and the precipitate was used as the Wharton’s jelly extracellular matrix (WJECM). The WJECM was placed in a container in which the upper portion of the liquid was in contact with the temperature gradient to guide the formation of crystals in the solution along the vertical temperature gradient. The mold and WJ scaffolds were prefrozen for approximately 16 h in a vacuum freeze dryer (Yonghao, 2XZ-2B, Linhai, China), after which the scaffolds were stored at −4 °C.

### Composite scaffold construction in vitro

The WJ scaffold was irradiated under 258 nm ultraviolet light and crosslinked for 8 h. For chemical crosslinking, the scaffolds were soaked for 30 min in a 40% ethanol solution supplemented with 50 mmol/L 2-morpholinoethanesulphonic (MES) acid and then soaked for 4 h in CS solution containing 40% ethanol, 24 mmol/L 1-(3-dimethylaminopropyl)-3-ethylcarbodiimide hydrochloride, 5.0 mmol/L N-hydroxysuccinimide and 50 mmol/L MES. The CS fraction was divided into three groups. In group A, the CS quality fraction was 1% (WJ + 1% CS); in group B, the CS quality fraction was 2% (WJ + 2% CS); and in group C, the CS quality fraction was 3% (WJ + 3% CS). The scaffolds were washed repeatedly in PBS, placed in a −80 °C freezer for 30 min and then placed into a vacuum freeze dryer, where they were dried again. After disinfection by ultraviolet rays, the samples were stored at 4 °C, and the composite scaffold structure was observed using scanning electron microscopy, hematoxylin and eosin (H&E) staining and toluidine blue (TB) staining; subsequently, the porosity, density, water absorption, degradation rate, and physical properties of the scaffolds were examined.

### Biomechanical testing of the scaffolds

The WJ scaffold and the 1% CS, 2% CS, and 3% CS composite scaffolds were shaped into cylinders (n = 5) with a diameter of 15.0 mm and a height of 6.0 mm. An Instron 3343 biomechanical testing instrument (Instron, 3343, USA) was used to evaluate the biomechanical performance of the scaffolds in axial compression. First, the scaffolds were placed in PBS to remain hydrated. After the mechanical testing software was opened, the compression method was selected, the mechanical sensors were connected, the preload was set to 0.05 N, and the scaffolds were placed between two rigid plates to ensure contact with the stent surface; the testing speed was 0.05 mm/s, and the measurement was stopped at a maximum load of 50 N. The magnitude of the reasonable force was determined according to the compression state. All tests were conducted within this range of reasonable forces. The experimental data in the test were automatically collected, and Young’s modulus was calculated from the slope of the stress–strain curve. Each experiment was performed three times, and the results were averaged.

### Cartilage defect in a rat model and implantation in vivo

The animal experiments were conducted according to the Animal Care and Use Committee of Taishan Medical University (No. 2017041). Male Sprague-Dawley (SD) rats (six weeks old) were selected for the cartilage defect model and subjected to anesthesia with an intraperitoneal injection of 0.1 ml/kg of 1% pentobarbital sodium. The rat knee joints were opened using a medial parapatellar approach. A full-thickness cylindrical cartilage defect with a diameter of 2 mm and a depth of 2 mm was introduced with a corneal trephine on the groove of the femur in both legs. Processing of the rat defect was divided into three groups: untreated control group (cartilage defect was not treated with a scaffold or hUCMSCs) (*n* = 10); WJ with hUCMSCs group (cartilage defect was treated with the WJ scaffold and hUCMSCs) (*n* = 10); and WJ + CS with hUCMSCs (cartilage defect was treated with the WJ + CS scaffold and hUCMSCs) (*n* = 10). After processing the different rat defects, the incision in the patella was closed by suturing, and an intramuscular injection of penicillin was given to prevent infection. During the postoperative period, the general condition of the animals was observed, and any sign of knee infection was noted. At 4 and 12 weeks, the animals were anesthetized with sodium pentobarbital and sacrificed; the joint cavity was opened, the knee joint was removed, and the repair state of the cartilage defects was observed followed by gross observations. Finally, histological analysis of the samples was performed.

### Gross observations in vivo

The surface texture, defect filling/size, and graft-recipient cartilage integration of the samples were grossly examined to evaluate the macroscopic structure of the repair tissue on the cartilage defects. The gross score was rated using a scale of 0–9, where high scores indicated degeneration and/or poor repair of the cartilage defects [23]. The scores of two blinded observers were averaged.

### Histological staining and scoring in vivo

The samples of repair tissue were fixed in 10% neutral-buffered formalin, decalcified in 10% EDTA, and paraffin embedded for histological analysis. Section 5 μm thick were cut, deparaffinized, and then stained with H&E, TB, safranin O and fast green according to standard procedures. All histological staining results were scored according to the histological scoring protocol described by Wakitani et al. [24]. The score ranged from 0–14 points, with 0 denoting complete regeneration and 14 denoting no regeneration. Thirty pathology images were selected, independently scored by three observers, and then averaged to increase the accuracy.

### Cell-derived detection of repaired cartilage in vivo

Human-specific ribonucleoprotein immunohistochemical staining (Chemicon, Temecula, CA, USA) was performed to determine the source of the hUCMSCs in the repair tissue in the cartilage defects of the three groups in vivo. The procedure described by Liu et al. [8] was followed [11, 12].

### Measurement of interleukin 6 (IL-6) in rat venous blood in vivo

Venous blood was obtained from rats of each group at 2 days, 1 week, 2 weeks, and 4 weeks after the operation and compared with that from nonoperative rats. The level of IL-6 was measured by enzyme-linked immunosorbent assay (ELISA). Venous blood samples were collected, and ethylenediaminetetraacetic acid disodium salt was added as an anticoagulant. After mixing for 10–20 min, the supernatant fluid was collected and centrifuged for 20 min (2000–3000 rpm/min). The reagents, samples, and standards in the ELISA kit (Langton Biotechnology Co., Ltd., Shanghai, China) for rat IL-6 were prepared according to the manufacturer’s requirements. The standards were prepared, and the biotin-labeled secondary antibody and ELISA reagents were added to the samples and incubated at 37 °C for 60 min. After the samples were washed 5 times, a color indicator was added and developed for 10 min at 37 °C, after which a stop solution was added. Within 10 min, the enzyme-labeled plate was placed into a standard microplate reader (Biotek, ELx800, Vermont, USA), and the absorbance was read.

### Statistical analysis

SPSS 19.0 and GraphPad Prism 6 statistical analysis software were used for the statistical analysis and plotting analysis. All experimental data are expressed as the mean ± standard deviation. The scores, contents of proteoglycan, acid mucopolysaccharide, and collagen as well as the mechanical properties and IL-6 ELISA data for each group were compared using variance analysis, and the least significant difference method was used to compare any pairwise difference; a difference of *P* < 0.05 was considered statistically significant.

## Results

### Characterization and surface markers of hUCMSCs in vitro

**Fig. 1:**
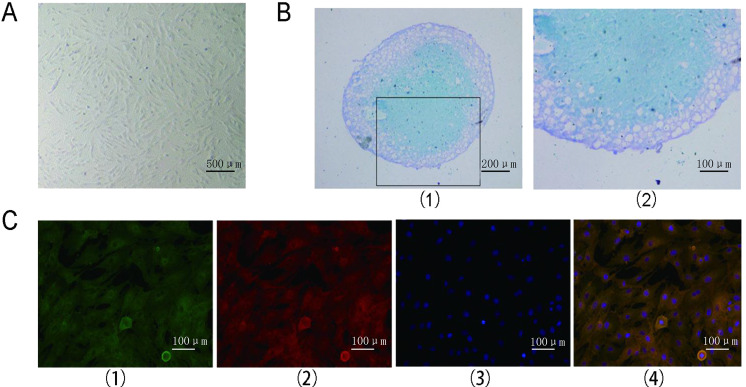
Isolation, culture and identification of hUCMSCs. **A** hUCMSCs were isolated from WJ and cultured for 7 days. Scale bar = 500 μm. **B** hUCMSCs were cultured and differentiated into chondrocytes for 21 days and then stained with alkaline blue. The cells around the cell mass that appear blue and round are chondrocytes rich in acid mucopolysaccharides, as shown in (1). Scale bar = 200 μm. (2) represents a portion of the enlarged box in (1). Scale bar = 100 μm. **C** (1) CD90 is shown by green fluorescence; (2) CD44 by red fluorescence; (3) the nucleus by blue fluorescence; (4) merge. Scale bar = 100 μm

The hUCMSCs were isolated from the WJ of UC tissue. After 7 days in primary culture, the adherent cells of the hUCMSCs could be observed. Under an inverted microscope, the cells appeared polygonal and spindle shaped, and cell proliferation was robust. After 2 weeks of culture, the cells were spindle shaped and arranged randomly (Fig. 1A). The hUCMSCs were cultured for 21 days. Next, 5-μm-thick paraffin sections were prepared and stained with alkaline blue; the blue color represents the acidic mucopolysaccharide of cartilage (Fig. 1B). The hUCMSCs were subjected to double immunofluorescence staining to detect CD44 and CD90 expression; CD44 staining is shown by red fluorescence, while CD90 staining is shown by green fluorescence; both showed positive results (Fig. 1C).

### Gross observation, histological observation, and physical properties of the scaffolds in vitro

**Fig. 2:**
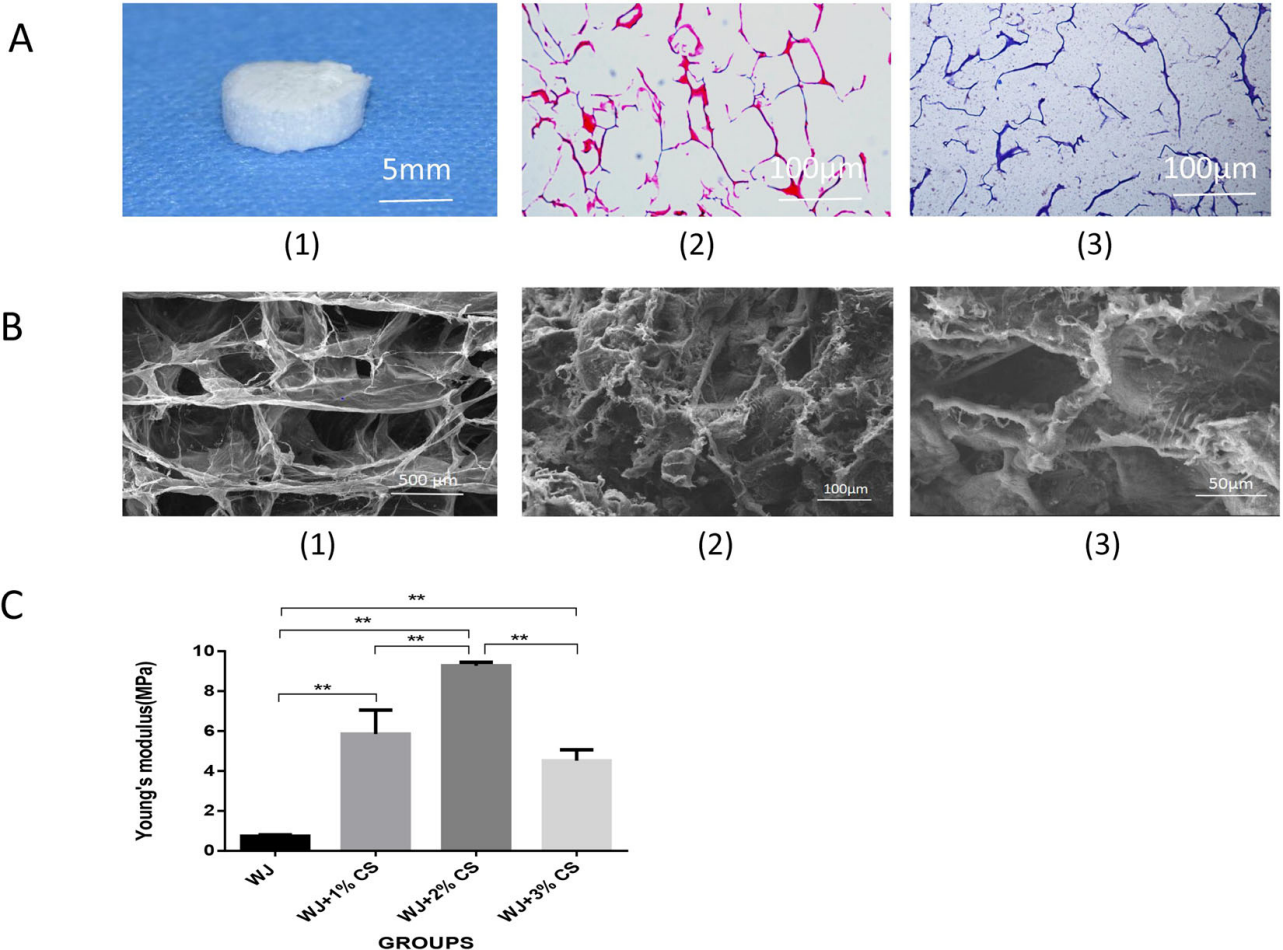
Scaffold test results. **A** (1) Gross observation of the scaffold: a lacunary, cylindrical and cavernous porous structure. Scale bar = 5 mm. (2) The WJ scaffolds were stained with H&E, and the low-magnification image shows multiple holes and a lattice-like network with a three-dimensional structure. Scale bar = 100 μm. (3) Scaffold with TB staining showing the secretion of proteoglycans. Scale bar = 100 μm. **B** (1) Longitudinal electron microscopy image of a scaffold, with the microtubules in an oriented structure. Scale bar = 500 μm. (2) The three-dimensional porous structure of the composite scaffold can be observed by electron microscopy. Scale bar = 100 μm. (3) Scale bar = 50 μm. **C** The Young’s modulus of the 2% composite group was the highest, followed by the 1% composite group, the 3% composite group and the WJ scaffold group; significant differences were found between the groups (**P < 0.01)

A white, cylindrical, sponge-like porous structure was observed in the scaffolds. The three-dimensional structure of the scaffold was macroscopically visible (Fig. 2A(1)), and H&E staining revealed that the scaffold had a porous, lattice-like three-dimensional structure (Fig. 2A(2)). Positive TB staining indicated proteoglycan secretion in the cartilage ECM (Fig. 2A(3)). Microscopically, the scaffold exhibited a three-dimensional porous structure with voids. Under a scanning electron microscope, the composite scaffold with a 2% mass fraction of CS combined with WJ showed a three-dimensional porous structure. The scaffolds were directional and arranged in parallel, and the interstices were interpenetrated. The microtubules were also oriented in a structure (Fig. 2B(1)–(3)). After the physical properties were determined, the porosities of the WJ scaffold and the composite scaffold were both found to be greater than 90%, and other physical features, such as density, water absorption and degradation rate, met the requirements of tissue-engineered scaffolds (Table 1).

**Table 1:** Physical performance of the scaffolds in each group

Group	Porosity (%)	Density (μg/mm^3^)	Water absorption rate (%)	Degradation rate (%)
WJ	91.13 ± 2.12	75.13 ± 6.22	1147 ± 85.71	20.69 ± 1.02
WJ + 1% CS	93.33 ± 1.11	82.33 ± 3.79	1287 ± 72.39	21.34 ± 1.73
WJ + 2% CS	92.33 ± 0.76	83.40 ± 2.92	1237 ± 56.04	21.24 ± 1.01
WJ + 3% CS	90.83 ± 1.46	79.11 ± 3.13	1036 ± 72.84	20.22 ± 2.02

### Biomechanical properties of the scaffolds in vitro

The axial compression biomechanical test showed that the Young’s modulus of the composite scaffold composed of different concentrations of CS was higher than that of the WJ scaffold (*P* < 0.05) (Fig. 2C). The Young’s modulus of the WJ + 2% CS composite scaffold was higher than that of either the WJ + 1% CS or WJ + 3% CS composite scaffold. The biomechanical performance of the WJ + 2% CS was the highest of all the composite scaffolds (Fig. 2C).

### Gross observation in vivo

**Fig. 3:**
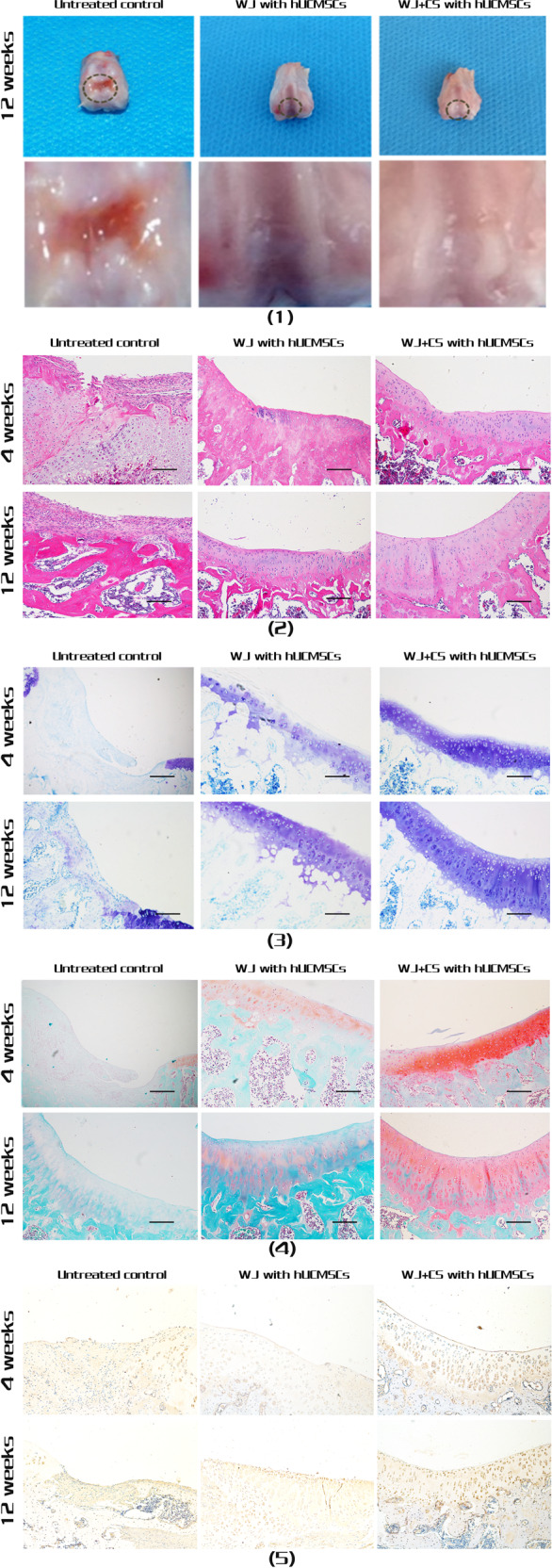
Gross observation and histological analysis of the cartilage defect repair. (1) Gross observation of the defect site in each group at 12 weeks after surgery. The lower figures are partially enlarged views of the upper figures. The defects of the untreated control group were basically filled, but the repair surface was still rough and poorly integrated with the surrounding normal cartilage. The repair surface of the WJ with hUCMSCs group was less smooth but integrated well with the surrounding normal tissue. The color of the repair tissue was very similar to that of the normal cartilage tissue in the WJ + CS with hUCMSCs group, and the flat repair surface integrated well with the surrounding normal tissue. (2) The images of cartilage repair defects include H&E (2), TB (3), safranin O (4) and type II collagen immunohistochemical staining (5). WJ + CS with hUCMSCs group: all stains in the WJ + CS with hUCMSCs group were positive. The repaired tissue was hyaline cartilage, and the arrangement of the chondrocytes was very similar to that in normal cartilage tissue. The repaired tissue integrated well with the surrounding normal tissue, and the wound was smooth and complete. The cartilage content in the extracellular matrix was very similar to that of the surrounding normal cartilage tissue; the type II collagen content of the repaired cartilage was similar to that of the normal cartilage. WJ with hUCMSCs group: the H&E, TB, safranin O and type II collagen immunohistochemical staining results were weakly positive; the repaired tissue was a mixture of fibrous tissue and hyaline cartilage, and the wound was not smooth; the cartilage content in the extracellular matrix was lower than that of the WJ + CS with hUCMSCs group; and the type II collagen content of the repaired tissue was low. Untreated control group: the H&E, TB, safranin O and type II collagen immunohistochemical staining results were weakly positive; the main repair tissue was fibrous tissue; the structure of the hyaline cartilage was difficult to detect in the defect site; the reconstructed tissue was poorly integrated with the surrounding normal tissue, and the wound was irregular; and the chondrocyte content in the extracellular matrix was the lowest. Scale bar = 100 μm

At 12 weeks after surgery, the cartilage defects of the untreated control group were basically filled, but the repair site was still rough, and integration with the surrounding normal cartilage was poor. The repair surface of the rat knee in the WJ with hUCMSCs group was less smooth but was well integrated into the surrounding normal tissue. In the WJ + CS with hUCMSCs group, the color of the repair tissue was close to that of the normal cartilage tissue, and the flat repair surface was integrated well into the surrounding tissue (Fig. 3(1)). Twelve weeks after surgery, the cartilage repair scores of the three groups were evaluated based on the surface features, degree of filling defect, and integration of the host grafts. The total scores of the untreated control, WJ with hUCMSCs and WJ + CS with hUCMSCs groups were 8.80 ± 0.45, 5.40 ± 1.14, and 4.00 ± 0.71, respectively. The *P* value indicates that the cartilage repair performance of the WJ + CS with hUCMSCs group was the best, followed by that of the WJ with hUCMSCs group, and the repair performance of the untreated control group was the worst (Table 2).

**Table 2:** Comparison of gross observation scores among the three groups ($\overline \chi \pm s$)

Group	*n*	Characteristics of the cartilage surface	Filling defect degree	Host graft fusion	Total score
Untreated control	5	2.80 ± 0.45	3.00 ± 0.00	3.00 ± 0.00	8.80 ± 0.45
WJ with hUCMSCs	5	2.00 ± 0.71^a^	1.00 ± 0.00^a^	2.40 ± 0.55^a^	5.40 ± 1.14^a^
WJ + CS with hUCMSCs	5	0.60 ± 0.55^ab^	2.20 ± 0.45^ab^	1.20 ± 0.45^ab^	4.00 ± 0.71^ab^
*F* value		18.600	76.000	25.200	45.700
*P* value		<0.001	<0.001	<0.001	<0.001

^a^Compared with the untreated control group, *P* < 0.05

^b^Compared with the WJ with hUCMSCs group, *P* < 0.05

### Histological observation and score in vivo

#### H&E staining

At 4 weeks after surgery, the repaired tissue of the untreated control group was mainly fibrous tissue, and numerous fusiform fibrillar cells were observed. The repaired tissue had obvious fissures, and the wound surface was uneven. The WJ with hUCMSCs group contained a mixture of fibroblasts and chondrocytes, the chondrocytes were unevenly distributed, and the wound surface was smooth and flat. The repaired tissue of the WJ + CS with hUCMSCs group was mainly hyaline cartilage, and many chondrocytes were observed. The arrangement of the cells in the repaired tissue was similar to that in normal cartilage tissue. The repaired tissue integrated well with the surrounding normal tissues; however, the wound surface was still uneven. At 12 weeks, the repaired tissue of the untreated control group was still fibrous tissue, the repair tissue of the WJ with hUCMSCs group was a mixture of fibrous tissue and hyaline cartilage, and the repaired tissue of the WJ + CS with hUCMSCs group was hyaline cartilage. The arrangement of the chondrocytes in the WJ + CS with hUCMSCs group was very similar to that in normal articular cartilage. The repaired tissue integrated well with the surrounding normal cartilage tissues, and the wound surface was smooth and complete (Fig. 3(2)).

#### TB staining

At 4 weeks after surgery, the untreated control group was negative for TB staining, the WJ with hUCMSCs group was weakly positive in a small area, and the WJ + CS with hUCMSCs group was strongly positive. At 12 weeks postsurgery, the repaired tissue in the untreated control group was still negative for TB staining, that in the WJ with hUCMSCs group was partially positive, and that in the WJ + CS with hUCMSCs group was strongly positive and very similar to the surrounding normal cartilage tissue (Fig. 3(3)). The results of the safranin O staining were similar to those of the TB staining (Fig. 3(4)).

#### Type II collagen immunohistochemical staining

Positive immunohistochemical staining for type II collagen is indicated by a specific brownish-yellow color. At 4 weeks after surgery, the repaired tissue of the untreated control group was negative for type II collagen, that of the WJ with hUCMSCs group was weakly positive for type II collagen, and that of the WJ + CS with hUCMSCs group was positive and similar to that of the surrounding normal tissue. At 12 weeks after surgery, the immunohistochemical staining of the repaired tissue of the untreated control group was still negative for type II collagen, that of the WJ with hUCMSCs group was weakly positive, and that of the WJ + CS with hUCMSCs group was strongly positive and similar to that of the surrounding normal tissue (Fig. 3(5)).

#### Histological score

**Fig. 4:**
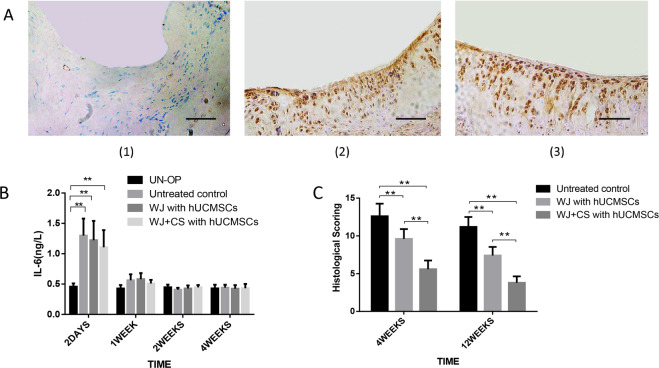
Immunogenicity and histological scoring in each in vivo transplantation group. **A** Immunohistochemical staining for an anti-human nucleoprotein at 12 weeks after surgery. The repaired cartilage in the untreated control group was stained blue, which indicates that the staining results were negative (1). The cartilage in the WJ with hUCMSCs and WJ + CS with hUCMSCs groups was stained brown, which indicates positive staining ((2) and (3)). Scale bar = 100 μm. **B** The histological scores of the WJ + CS with hUCMSCs group at 4 and 12 weeks were lower than those of the WJ with hUCMSCs and untreated control groups (*P* < 0.05). The histological scores of the WJ + CS with hUCMSCs group were the lowest at each time point (*P* < 0.05). **C** The IL-6 levels in all surgery groups were significantly higher than those in the unoperated (UN-OP) groups 2 days after surgery (*P* < 0.05). At 1, 2 and 4 weeks, the IL-6 levels in the untreated control, WJ with hUCMSCs, and WJ + CS with hUCMSCs groups were equal to those in the unoperated group, and no significant difference was observed between the groups (*P* > 0.05)

According to the rules of the Wakitani law histology score, the lower the score is, the better the effect. The histological scores of the WJ + CS with hUCMSCs group at 4 and 12 weeks were lower than those of the WJ with hUCMSCs and untreated control groups (*P* < 0.05). The histological scores of the WJ + CS with hUCMSCs groups were lowest at each time point (*P* < 0.05) (Fig. 4C).

#### Cell-derived detection of repaired cartilage

The source of the repaired chondrocytes was identified by immunohistochemical staining for an anti-human nucleoprotein, which was performed 12 weeks after surgery. The staining results showed that the untreated control group was negative for this nucleoprotein, and both the WJ with hUCMSCs and WJ + CS with hUCMSCs groups were positive and fused well with the surrounding tissues (Fig. 4A).

### In vivo immunogenicity test

The IL-6 levels in all surgery groups were significantly higher than those in the unoperated groups at two days after surgery (*P* < 0.05). At 1, 2 and 4 weeks, the IL-6 levels in the untreated control, WJ with hUCMSCs, and WJ + CS with hUCMSCs groups were equal to those in the unoperated groups, and no significant difference was observed between the groups (*P* > 0.05) (Fig. 4B).

## Discussion

In this study, we used hUCMSCs as seed cells because they have the following advantages: 1. abundant source; 2. no pain caused by secondary trauma to the donors; 3. excellent plasticity; 4. strong amplification ability; 5. low immunogenicity; 6. proliferative ability and differentiation ability that will not decrease with increased passaging or age; and 7. paracrine function [25–28]. In this experiment, hUCMSCs were successfully isolated from human UCs. An immunofluorescence double staining method was used to show that the isolated cultured hUCMSCs can simultaneously express CD44 and CD90, which indicates that they exhibit characteristics of MSCs. The hUCMSCs underwent cartilage-induced differentiation for 21 days, and the detection of cartilage-specific components was positive, which indicates that the hUCMSCs had chondrogenic potential. The hUCMSCs in this study demonstrated a good repair effect on cartilage defects. The ELISA results showed increased IL-6 levels after surgery due to traumatic stress, but they returned to normal levels after 1 week and did not increase or fluctuate within 4 weeks. This phenomenon indicates that no immunological reaction occurred during transplantation. Immunohistochemical staining for an anti-human nuclear protein 12 weeks after surgery showed that the repaired cartilage cells were derived from the hUCMSCs. Therefore, hUCMSCs have low immunogenicity for transplantation in tissue-engineered cartilage.

As a highly organized tissue, articular cartilage is driven by the zonal heterogeneity of cells, extracellular matrix proteins and fibril orientations, which result in depth-dependent mechanical properties [29]. Therefore, reproducing the functional properties of natural cartilage in tissue-engineered constructs requires the consideration of mechanical properties. Although researchers have recently studied the mechanical properties of cartilage tissue-engineered scaffolds [30, 31], a challenge remains. The ideal scaffold should have many specific architectural, physicochemical, biological, and mechanical properties [32, 33]. Cartilage tissue-engineered scaffold materials are classified mainly into natural materials and synthetic materials. Natural materials have good biocompatibility, easy degradation, relatively low toxicity, easy absorption, and low inflammatory reactions, but they also have many shortcomings, such as poor mechanical strength and difficulty in being controlled [15]. For cartilage tissue-engineered scaffolds, increasing numbers of scholars have focused on decellularized cartilage matrix for the construction of engineered cartilage tissue. UC-derived WJ, a natural material, has a similar composition and biological functions as cartilage matrix, low immunogenicity, and the ability to inhibit immune rejection and induce host immune tolerance [8].

The hWJECM is abundant and had similar biochemistry to the cartilage ECM, and its use is not associated with ethical controversy. The three-dimensional scaffold has a porous and well-oriented structure, with a mean pore diameter of 104 μm. Scanning electron microscopy and cell viability staining results demonstrated that the oriented scaffold has good biocompatibility and cell alignment [15]. Moreover, they showed good water uptake ratios and compressive moduli [17]. Since the scaffold material composed of WJ is similar to a sponge and has relatively poor mechanical properties, we optimized the scaffold by physical and chemical crosslinking and manufactured a novel composite scaffold based on the WJ scaffold. For the optimum ratio of CS to scaffold, we found that the composite scaffold containing a residual 2% mass fraction of CS achieved the best porosity and had a higher Young’s modulus than the other composite groups according to the in vitro biomechanical test (Table 1). The prepared composite scaffold had a moderate pore size and high porosity and was suitable for seed cell growth. We found that the increased biomechanical properties resulted from the support of the oriented structure in the composite scaffold, which played an important role after CS was added; a similar phenomenon was observed in previous studies [34]. Therefore, WJ + 2% CS was used as the basis for subsequent in vivo experiments.

For the in vivo experiment, the rats were divided into three groups: untreated control group, WJ with hUCMSCs group, and WJ + CS with hUCMSCs group. The purpose was to determine whether the composite scaffold with good mechanical properties had better repair effects on cartilage defects than the control group without cells and the WJ scaffold group. The scaffold was directly used to repair knee articular cartilage defects in SD rats. After the longest follow-up at 12 weeks, the gross observation results showed that the tissue repaired by the composite scaffold + hUCMSCs was most similar to normal cartilage and was superior to tissue repaired by the hUCMSC-loaded WJ scaffold and the untreated control, as shown by gross observation and histologic analysis. The hWJMSC-loaded composite scaffold repaired articular cartilage defects because the structural and physical properties of the WJ scaffold were changed by the physical and chemical crosslinking process after the scaffold was combined with 2% CS. First, the orientation of the composite scaffolds was beneficial to the growth and differentiation of hUCMSCs. Second, the antipressure performance of the composite scaffold was improved due to the change in its orientation, which overcame the shortcomings of the mechanical properties of the WJ scaffold and supported and protected the hUCMSCs. Third, the low immunogenicity of the composite scaffold prevented immunological reactions to the cartilage defects of the rat knee after transplantation. As seen from the repair tissue stained for an anti-human nucleoprotein in cartilage defects of the rat knee, the WJ + CS with hUCMSCs group and the WJ with hUCMSCs group were positive, while the untreated group was negative, which indicates that the cell source of the repaired tissue was directly related to hUCMSCs. In addition, the hUCMSC scaffold achieved better quality repair and regeneration of hyaline cartilage without cartilage-inducing factors while retaining the structure and functional integrity of the subchondral bone [18].

Regarding the immune response, Il-6 test results of the venous blood of transplanted animals revealed no significant difference between the composite scaffold + hUCMSCs group, the WJ scaffold + hUCMSCs group and the untreated group at 2, 4 or 12 weeks after surgery. These results indicated no immune rejection of the composite scaffold and hUCMSCs, as both the scaffold and hUCMSCs showed low immunogenicity. The deficiencies of this experiment are as follows: we are not able to prove the nonmineralization ability of the scaffolds, the quantitative parameters of cell proliferation (e.g., DNA) or the viability of cells due to the limitations of experimental materials and scientific researchers. The tissue specimens were selected from the cartilage tissue of SD rat knees, and whether the repair effect on the rat cartilage in this study could be observed in other large animal models or even in clinical applications needs to be verified in future experiments. The mechanism of repair of articular cartilage defects by hUCMSCs combined with a scaffold should therefore be further explored.

## Conclusions

Our research shows that hUMSCs have chondrogenic potential, and the composite scaffold comprised of WJ and CS, which exhibits a three-dimensional porous structure, good physical properties, mechanical properties and biocompatibility. In vivo experiments in SD rats showed that the tissue repaired by the composite scaffold + hUCMSCs was hyaline cartilage, and the arrangement of the chondrocytes was very similar to that in normal cartilage tissue. The repaired tissue integrated well with the surrounding normal tissue, and the wound was smooth and complete. Therefore, the composite scaffold + hUCMSCs are very promising in the repair of articular cartilage defects. They can significantly improve the repair effect of cartilage defects. It also provides a new option for cartilage tissue scaffold materials.
